# Targeting NFκB signaling in GD2^+^ BCSCs

**DOI:** 10.18632/aging.101274

**Published:** 2017-08-03

**Authors:** Khoa Nguyen, V. Lokesh Battula

**Affiliations:** Section of Molecular Hematology and Therapy, Leukemia Department & Department of Breast Medical Oncology, UT MD Anderson Cancer Center, Houston, TX, 77030, USA

**Keywords:** breast cancer stem cells, BCSC, GD2, Triple negative breast cancer, NFκB signaling, BMS345541, ST8SIA1, GD3 synthase

Breast cancer stem-like cells (BCSCs) have recently become a research priority because of their ability to initiate tumors, cause metastasis, and resist chemo-therapeutic drugs. Although BCSCs make up a small fraction of the bulk of tumor cells, their unique characteristics allow them to mitigate the effects of chemotherapy and cause cancer recurrence. In order to develop a therapy to specifically target BCSCs, an identification method must be used to discriminate them from the bulk tumor. Michael Clark's group identified CD44^High^CD24^Low^ expression unique to the stem-like population of the heterogeneous tumor [1]. This widely recognized phenotype is currently being used by investigators to identify and isolate BCSCs from primary tumors. In 2012, we discovered that gan-glioside GD2 identifies BCSCs and is expressed exclusively in the CD44^High^CD24^Low^ fraction of Triple Negative Breast Cancer (TNBC) cells. Additionally, GD2^+^ cells are highly tumorigenic in-vivo [2]. GD2 is a cell surface ganglioside that is synthesized from its precursor, GD3, by GD2 synthase. However, gene expression analysis revealed that GD3 synthase (ST8SIA1) but not GD2 synthase is upregulated in GD2^+^ cells compared to GD2^−^ cells; suggesting that GD3S is the rate limiting factor for GD2 expression in BCSCs. Knocking out GD3S dramatically inhibited tumor growth and metastasis of TNBC cell lines in-vivo. Additionally, reports from other groups suggest that GD3S is highly expressed in patient derived BCSCs. These findings suggest that factors that regulate GD3S expression may be an ideal candidate for targeted therapy in TNBC.

Activation of NFkB signaling has been well described in several diseases including TNBC [3], however, its role in BCSCs function is not well understood. NFκB signaling mainly consists of two parts including canonical and non-canonical pathways which transmit signals to regulate downstream targets upon ligand mediated receptor activation. As a part of the canonical NFkB pathway, RelA translocates into the nucleus to induce gene expression when its inhibitor, IkB, gets phosphorylated by a heterodimer composed of IKKα and IKKß. Alternatively, the non-canonical pathway phosphorylates IkB with an IKKß independent IKKα homodimer resulting in nuclear translocation of RelB. Using antibody array technology, we recently found that the protein p65 (RelA) is highly activated in the GD2^+^ BCSCs compared to the GD2^−^ in TNBC cell lines [4]. Knocking down IKKα, the regulator of both the canonical and non-canonical pathways, inhibited GD3S expression. More importantly, it also reduced the percentage of GD2^+^ BCSCs [4] suggesting that NFkB signaling regulates BCSC growth in TNBC cells. Next, we tested several small molecule inhibitors targeting NFkB signaling at different stages and identified that BMS345541 (Inhibitor of IKKβ at IC50 0.4μM and IKKα at IC50 4μM) inhibits GD2 and GD3S expression in TNBC cells [4]. As it targets IKK proteins, BMS-345541 interrupts both the canonical and non-canonical pathway by thwarting the phosphorylation of IkB and thus preventing RelA (canonical) and RelB (non-canonical) from translocating across the nuclear membrane.

In-vitro, BMS-345541 reduced the absolute number of GD2^+^ cells in a dose and time dependent manner in TNBC cell lines SUM-159 and MDA-MB-231[4]. In addition to the phenotypic changes, BMS345541 also inhibited GD3S (which is a rate limiting factor for GD2 expression) mRNA expression in a concentration dependent manner [4]. To test whether or not stem cell activity was truly impacted, we performed a series of functional assays to test how inhibition of NFkB signaling would affect BCSCs. When cultured under low-adherent conditions, MDA-MB-231 and SUM-159 cell lines showed a reduction in mammosphere formation when treated with BMS-345541. When cultured in a 3D gel matrix, cells exposed to higher concentrations of BMS-345541 formed smaller and lower number of colonies compared to their untreated control counterparts [4]. Moreover, BMS-345541 reduced cell migration and invasion by approximately 10-fold and 40-fold in TNBC cell lines. In-vivo, tumor growth and lung metastasis was greatly affected as well. Mice treated with BMS-345541 tended to show significant tumor growth reduction compared to the control [4]. In addition, metastatic mice had smaller and fewer metastatic lung colonies compared to the control group[4] suggesting targeting NKκB signaling inhibits GD2+ BCSC growth and thereby inhibits TNBC tumor growth and metastases.

Nuclear Factor-Kappa B is a protein complex that controls DNA transcription, cytokine production, and cell survival. Its biological role ranges from immune responses to nervous learning and plasticity. Because of its role in presence in many different cell types, disruption of NFkB signaling can lead to many different diseases and disorders with carcinogenic tendencies. Other groups have shown NFkB signaling to be a major pathway in breast cancer with some suggesting it as a therapeutic target for different cancer subtypes [5, 6]. Additionally, it has been reported that NFkB has plays a role in regulating BCSCs by promoting stem cell self-renewal [7]. In our paper, *IKK inhibition by BMS-345541 suppresses breast tumorigensis and metastases by targeting GD2^+^ cancer stem cells*, we show that NFkB plays a key role in BCSC formation and function in TNBC by correlating its activity with GD2^+^ expression[4]. By using the IKK inhibitor, BMS-345541, we were able to abrogate the deadly effects of cancer stem cells in both in-vitro and in-vivo settings. Our data supports the idea that NFkB mediated signaling is up-regulates GD2^+^ BCSCs by regulating GD3S expression. BMS-345541, an inhibitor for IKK proteins in the NFkB pathway, interferes with stem cell genesis, tumor growth and metastasis in TNBC. BMS-345541 has the potential to treat metastatic TNBC when used in combination with conventional chemotherapy.
